# A case of emphysematous cystitis caused by mechanical stimulation of pelvic fracture nonunion

**DOI:** 10.1016/j.tcr.2024.100987

**Published:** 2024-03-12

**Authors:** Yasuaki Yamakawa, Yasutaka Masada, Ryo Ugawa, Tadashi Komatsubara, Kunihiko Numoto, Toshiyuki Mastumoto

**Affiliations:** Department of Orthopedic Surgery, Kochi Health Sciences Center, Kochi, Japan

**Keywords:** Pelvic fracture, Emphysematous cystitis, Diabetes mellitus, Urinary tract infections, Complications

## Abstract

Emphysematous cystitis is a rare condition that develops due to tissue hyperglycemia and urinary tract infection by gas-producing bacteria. We report a case of emphysematous cystitis caused by mechanical stimulation of a pelvic fracture nonunion.

An 80-year-old man was injured in a motorcycle accident and diagnosed with pelvic fracture. Seven days later, he had high fever and computed tomography (CT) revealed gas in the hematoma around the pelvic fracture and the abscess. Therefore, infection following the pelvic fracture was diagnosed. Despite multiple operations and antibiotics treatment, malformation and nonunion of the pelvis occurred. One month after starting weight bearing, emphysema of the bladder wall adjacent to the pubic fracture were found and spread throughout the bladder wall. With stopping of weight bearing, antibiotics treatment and a urinary catheter, emphysema disappeared after 2 months.

It was considered that the pubic fracture fragment irritated the bladder wall due to weight bearing and emphysematous cystitis consequently developed.

## Introduction

Although there are many reports about bladder injuries associated with pelvic fractures, most of them occur at the time of injury, and there are only a few case reports of delayed bladder-related complications [1,2].

Emphysematous cystitis is one of emphysematous urinary tract infections and can be fatal if it is severe. Diabetes mellitus and urinary tract infection are considered to be a risk factor. The mechanism was thought to as gas productive bacteria ferment and decompose the glucose within peri-urinary tissues and urine, and result in emphysema cystitis [3,4]. In addition, there are no reports of emphysematous cystitis associated with pelvic fractures. We report a case of emphysematous cystitis caused by the mechanical stimulation of the pelvic fracture nonunion and discussed possible mechanism.

## Case presentation

### Patient history of admission

**Fig. 1:**
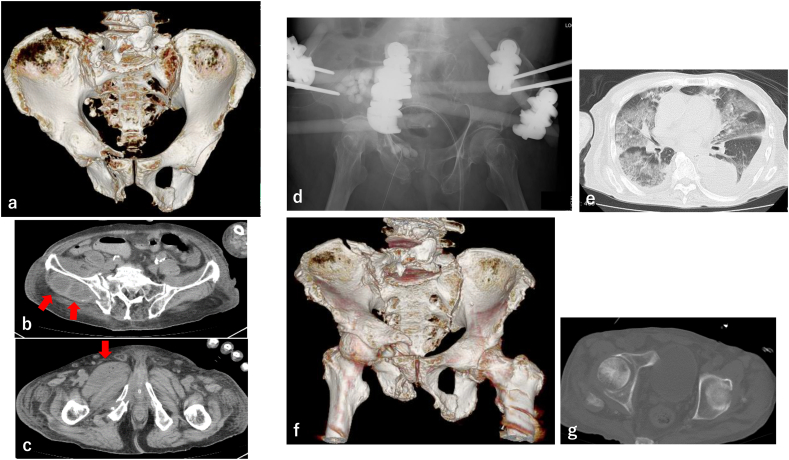
Radiographs and computed tomography (CT) of the pelvis and lung (a) Three-dimensional (3D) CT shows right iliac fracture, sacroiliac joint dislocation, and right pubic fracture classified to AO/OTA classification 61C1.2 and (b, c) abscess formation in the right hip and adductor muscle (red arrow). (d) Plain radiograph after continuous local antibiotics perfusion (CLAP), antibiotic-impregnated cement implantation, and external fixation of the pelvic fracture. (e) Bilateral infiltrates, indicative of eosinophilic pneumonia. (f) 3DCT of the pelvis 2 months after injury show deformation and nonunion and (g) no emphysematous cystitis. (For interpretation of the references to colour in this figure legend, the reader is referred to the web version of this article.)

80-year-old male had injured while riding a motorcycle. Three days later, he consulted the previous doctor and was admitted to the hospital with a diagnosis of right pubis fracture. Seven days after admission, he developed fever and the hematoma including gas around the pelvic fracture was observed on computed tomography (CT). He had been diagnosed as infection of pelvic fracture and then transported to our hospital. CT scan at the time of the visit revealed that the fracture of the right ilium and sacrum had become apparent and increased displacement (Fig. 1a), which were classified to AO/OTA classification 61C1.2, and the fluid was observed around the right gluteal and adductor muscles (Fig. 1b, c). MSSA (Methicillin-Susceptible *Staphylococcus Aureus*) was detected from a puncture culture of the right buttock.

His general condition was too bad, so multidisciplinary treatment was performed in the intensive care unit. MSSA was also detected in urine culture, and the relationship between pelvic fracture and urine infection was suspected. A cystoscopy was performed 7 days after admission, but no obvious injury lesions of the bladder or urethra were found. A follow-up CT scan showed an enlargement of abscess around right gluteal and the right pubic, adductor muscles. Multimodal treatment and debridement of the abscess improved the general condition, but the local infection around the pelvic fracture persisted. At 35 days after admission, continuous local antibiotics perfusion (CLAP), antibiotics including cement beads were used for infection and external fixation of the pelvis were performed to stabilize the pelvic fracture (Fig. 1d). CLAP was continued for 10 days and the external fixation was removed after 3 weeks. At the same time, pneumonia and eosinophilia (maximum fraction 17.8 %) were observed, and mini pulse of prednisolone was started under the diagnosis of eosinophilic pneumonia (Fig. 1e). The pain decreased over time, and the patient became able to use the wheelchair, but the pelvic fracture became malformation and nonunion (Fig. 1f, g). Moreover, with his cognitive deterioration, additional surgical treatment such as deformity correction or internal fixation was avoided. Even the urinary tract infection was also persisted, the patient was transferred to a rehabilitation hospital 78 days after admission.

### Course after discharge

**Fig. 2:**
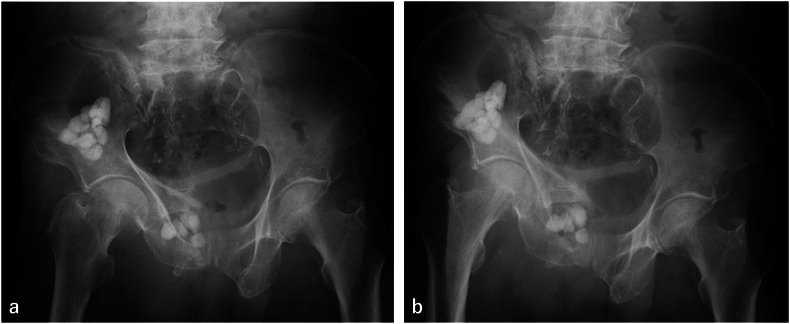
Plain X-ray photograph of the pelvis under push-pull test (a) The right lower limb is pulled caudally and the left lower limb is pulled cranially. (b) Pulled to the opposite side of (a). There is no obvious instability.

**Fig. 3:**
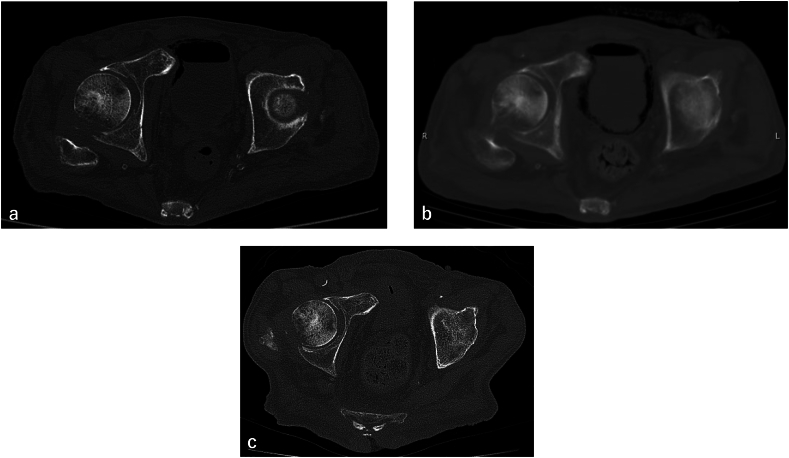
Serial computed tomography (CT) of the pubic nonunion fracture site and bladder (a) Emphysema of the bladder wall in contact with the pubic nonunion site is seen after starting weight bearing. (b) Emphysema has spread over the bladder wall in 2 weeks. (c) Emphysema has disappeared after 2 months.

At six months after the injury, stress radiography of the pelvis was performed. It was judged that there was no instability of the fractured site (Fig. 2a, b), and weight bearing was started. About one month after starting weight bearing, hematuria appeared, and CT confirmed emphysema in the bladder wall adjacent to the pubic fracture (Fig. 3a). Two weeks later, the emphysema progressed in the entire bladder wall (Fig. 3b), and he was returned to our hospital. *Escherichia coli* (ESBL) was detected from the urine culture. He was diagnosed as emphysematous cystitis caused by stimulation of the pubic nonunion fragment associated with weight bearing and urinary tract infection. Conservative treatment by indwelling urethral catheter, antibiotic treatment (tazobactam/piperacillin), and non-weight bearing was performed. Two months after the onset of emphysematous cystitis, emphysema has disappeared (Fig. 3c).

## Discussion

Emphysematous cystitis was first reported as “Cystitis emphysematosa” by Bailey in 1961 [4]. With advances in diagnostic imaging tools, the report of emphysematous cystitis has been increasing [3]. As epidemiological risk factors for emphysematous cystitis, women and diabetes mellitus have been reported [3,5]. As the mechanism of emphysematous cystitis, the combination of gas productive bacteria associated with urinary tract infection, hyperglycemia in local tissues associated with diabetes, and insufficient tissue metabolism are thought [6]. In this case, although the patient was a male and no diabetes mellitus was observed on admission, there was a possibility that he had been using steroids for eosinophilic pneumonia during the course of his treatment, resulting in latent tissue hyperglycemia. In addition, the presence of persisted urinary tract infections was thought to play a role.

There were no reports of emphysematous cystitis occurred by mechanical stimulation of pelvic fractures. As for the possible mechanism of this case, although no obvious instability of the fractured site was observed in pelvic stress radiograph six months after the injury, it was thought that the slight movement and stimulation of the pubic nonunion fragment caused by the weight bearing have contributed to the development of the emphysematous cystitis. It is possible that a small perforation on the serosa side of the bladder wall may have been the trigger of emphysema, or that repeated mechanical stimulation, even without a small perforation, may have caused a vacuum phenomenon within the bladder wall [7]. After the onset of emphysema, emphysema spread throughout the bladder over time, and it is also presumed that prolonged urinary tract infection and latent tissue hyperglycemia might contributed to the development of emphysematous cystitis. These mechanisms were not confirmed, however, follow-up CT scans showed that there was no emphysema around the bladder before the start of weight bearing, emphysema has occurred from adjacent to the pubic fracture and spread overall finally. The fact that emphysematous progression after the onset of weight-bearing strongly suggests that the mechanical stimulation of the pubic fracture nonunion was involved in the onset of emphysematous cystitis. To prevent this complication, continuing non-weight bearing or surgical correction or stabilization for pelvic deformity would be choice.

As for the cause of the infection around the pelvic fracture at the time of the injury, urethral or bladder injury was not evident even after cystoscopy. However, the fact that the same MSSA was detected in gluteal abscess and urine cultures was considered to be related. It is possible that the pubic bone fragment displaced medially at the time of injury caused bladder injury, and the infection spread from the urinary tract infection to the area around the pelvic fracture via the bladder injury. The caution is required for the bladder complications related to pelvic fracture at any time, during acute and even chronic phase, although it may be limited to certain conditions like pelvic fracture nonunion in the chronic phase.

## Conclusion

We reported a case of emphysematous cystitis, which was presumed to be caused by mechanical stimulation of the pelvic fracture nonunion. Bladder complications associated with pelvic fractures occur not only in the acute phase, but also in the chronic phase when the pelvic fracture become nonunion.

## Funding

None.

## CRediT authorship contribution statement

**Yasuaki Yamakawa:** Conceptualization, Investigation, Writing – original draft, Writing – review & editing. **Yasutaka Masada:** Writing – review & editing. **Ryo Ugawa:** Writing – review & editing. **Tadashi Komatsubara:** Writing – review & editing. **Kunihiko Numoto:** Writing – review & editing. **Toshiyuki Mastumoto:** Writing – review & editing.

## Declaration of competing interest

None.
